# Requirement of Cellular Protein CCT7 for the Replication of Fowl Adenovirus Serotype 4 (FAdV-4) in Leghorn Male Hepatocellular Cells Via Interaction with the Viral Hexon Protein

**DOI:** 10.3390/v11020107

**Published:** 2019-01-27

**Authors:** Junfeng Gao, Mingliang Zhao, Xueyan Duan, Yongqiang Wang, Hong Cao, Xiaoqi Li, Shijun J. Zheng

**Affiliations:** 1 Key Laboratory of Animal Epidemiology of the Ministry of Agriculture, China Agricultural University, Beijing 100193, China; jfgao@cau.edu.cn (J.G.); brightzhao1992@163.com (M.Z.); bestxy@188.com (X.D.); vetwyq@cau.edu.cn (Y.W.); caohong@cau.edu.cn (H.C.); 2 College of Veterinary Medicine, China Agricultural University, Beijing 100193, China

**Keywords:** FAdV-4, Hexon, CCT7, Replication

## Abstract

Fowl adenovirus serotype 4 (FAdV-4) causes hepatitis-hydropericardium syndrome (HHS), leading to severe economic losses in the poultry industry. Although the pathogenesis of FAdV-4 infection has caused much attention, the underlying molecular mechanisms remain poorly understood. Here, we identified chaperonin containing TCP-1 subunit eta (CCT7) as an interacting partner of the FAdV-4 capsid protein hexon. We found that ectopic expression of CCT7 in leghorn male hepatocellular (LMH) cells enhanced hexon expression in pRK5-flag-hexon transfected cells. On the contrary, knockdown of cellular CCT7 by RNAi markedly reduced hexon expression in FAdV-4-infected cells and suppressed viral replication. These data suggest that CCT7 is required for FAdV-4 replication and may serve as a potential target for controlling FAdV-4 infection.

## 1. Introduction

Fowl adenovirus serotype 4 (FAdV-4), a member of the *Aviadenovirus* genus in the *Adenoviridae* family, is an important pathogen of chickens, causing hepatitis-hydropericardium syndrome (HHS) and leading to significant risk in the poultry industry [1,2]. HHS was initially reported in Pakistan in 1987, and subsequently broke out in South America and Asia, including Iraq [3], Japan [4], Chile [5], Korea [6], and China [7,8]. The gross lesions in FAdV-4-infected birds are characterized by a hydropericardium and a swollen and yellow brown-colored liver with foci of hemorrhages and necrosis [2,9]. FAdV-4 is an icosahedral nonenveloped virus with a capsid shell containing a linear and non-segmented double-stranded DNA (dsDNA) [10]. Its genome encodes 10 major structural proteins in the virion, including hexon; penton base; fiber-1; fiber-2; terminal protein; and proteins Ⅵ, Ⅶ, Ⅷ, Ⅲ, and μ [11]. It was found that hexon and fiber-2 play critical roles in FAdV-4 pathogenicity by using a reverse genetics system [12]. Recombinant FAdV-4 fiber-2 has been identified as a protective antigen against HHS in chickens [13,14]. In the mammalian humoral immune responses to adenoviruses, the antibodies against hexons and fibers account for most of the neutralizing activity [15,16]. 

T-complex polypeptide 1 subunit eta (TCP1 eta, CCT7, CCTη) is a cytosolic chaperone protein that belongs to the eukaryotic chaperonin T-complex protein-1 (TCP-1) ring complex (TRiC) [17]. TRiC is a large complex of ~900kDa formed by two eight-membered rings composed of different subunits (CCT1-CCT8) [18]. It has been found that TRiC can help the folding of α-actin [19], peroxisome membrane protein Pmp22 [20], cdc20 [21], pG-protein β subunits [22], and von Hippel-Lindau tumor-suppressor protein [23]. Recent evidence shows that TRiC takes part in the regulation of viral infection [24,25]. It has been reported that influenza virus RNA polymerase subunit PB2 is associated with CCT as a monomer and silencing of CCT resulted in the reduction of viral RNA accumulation [26]. The host protein CCTγ is associated with Negri bodies in rabies virus (RABV)-infected N2a cells and contributes to RABV genomic replication [27]. TRiC can form a complex with the reovirus σ3 outer-capsid protein and folds σ3 into its native conformation [28].

Although FAdV-4-infection causes severe inflammatory response and induces target organ damage [29,30], the underlying mechanism of FAdV-4 infection is largely unknown. In this study, we analyzed the binding partners of FAdV-4 hexon in leghorn male hepatocellular cells by a liquid chromatography-mass spectrograph-based proteomic approach and identified a crucial cellular protein CCT7 associated with the replication of FAdV-4.

## 2. Materials and Methods

### 2.1. Virus and Cells

FAdV-4 HuBWH strain was isolated from the liver of HHS-affected chicken in Wuhan areas of China in 2016. The isolate was further purified by plaque forming unit assay (PFU). LMH, an immortalized chicken liver cell line, was kindly provided by Dr. Jinhua Liu (CAU, Beijing, China). The cells were cultured in Waymouth’s Medium (M&C Gene Technology, Beijing, China) supplemented with 10% fetal bovine serum (Gibco, San Diego, CA, USA) in a 5% CO_2_ incubator. HeLa cell line was obtained from ATCC, grown in Dulbecco’s modified Eagle’s medium (DMEM) (Invitrogen, Carlsbad, CA, USA) supplemented with 10% fetal bovine serum in a 5% CO_2_ incubator.

### 2.2. Reagents

All restriction enzymes were purchased from TaKaRa (Kusatsu, Shiga, Japan). The pRK5-FLAG, pCMV-Myc, pDsRed-monomer-N1 and pEGFP-C1 vectors were obtained from Clontech. Endotoxin-free plasmid preparation Kits were purchased from Magen (Guangzhou, China). Protein A/G plus-agarose was purchased from GE Healthcare Life Sciences (Uppsala, Sweden). Anti-GAPDH monoclonal antibody was obtained from GBC lifetech Company (Beijing, China). Anti-FAdV-4 hexon monoclonal antibody and anti- FAdV-4 hexon polyclonal antibody were obtained from CAEU Biological Company (Beijing, China). CCT7 polyclonal antibodies (A12146) were purchased from ABclonal Technology (Wuhan, China). Myc-Tag mouse mAb (2276) was purchased from Cell Signaling Technology (Danvers, MA, USA). Anti-FLAG M2 (F1804) antibody was purchased from Sigma Aldrich (St. Louis, MO, USA). FITC-conjugated goat anti-mouse IgG, TRITC-conjugated goat anti-mouse IgG, horseradish peroxidase (HRP)-conjugated goat anti-mouse and anti-rabbit IgG antibodies were purchased from DingGuoShengWu (Beijing, China). DyLight 488 AffiniPure goat anti-rabbit IgG antibody was purchased from Abbkine (Redlands, CA, USA). The jetPRIME transfection reagent (114-01) was purchased from Polyplus-transfection (Strasbourg, France). 4′,6-Diamidino-2-phenylindole (DAPI) was purchased from Beyotime (Nanjing, China). Protease inhibitor cocktail C was obtained from YTHX Biotechnology Company (Beijing, China). An enhanced chemiluminescence (ECL) kit was purchased from Merck Millipore (Darmstadt, Germany). 

### 2.3. Constructs

The FAdV-4 hexon was cloned from a FAdV-4 HuBWH strain using specific primers (sense primer 5′-ATG GCG GCC CTC ACG CCC GA-3′ and antisense primer 5′-TTA CAC GGC GTT GCC TGT GG-3′) according to the sequence in GenBank (accession number KU991797.1). Chicken cct7 gene was cloned from the cDNA of LMH cells using specific primers (sense primer 5′-ATG ATG CCC ACA CCG GTT ATCC-3′ and antisense primer 5′-TCA GTG GTT GTG GGG TCT GCCC-3′ [GenBank accession number NM_001031541.2]). PRK5-flag-hexon, pEGFP-C1-hexon, pDsRed-cct7, pCMV-Myc-cct7 and truncated pCMV-Myc-cct7 (Δ1: 1-136aa; Δ2: 1-272aa; Δ3: 1-408aa; Δ4: 137-544aa; Δ5:273-544aa; Δ6: 409-544aa) expression plasmids were constructed by standard molecular biology techniques. All the primers were synthesized by Sangon Company (Shanghai, China).

### 2.4. Pull-Down Assay

LMH cells were seeded on 100-mm cell culture dishes and cultured for 24 h before transfection with pRK5-FLAG-hexon using jetPRIME^®^ transfection reagent or infected with FAdV-4. Twenty-four hours after transfection, cell lysates were prepared using a nondenaturing lysis buffer (50 mM Tris-HCl, pH 8.0, 150 mM NaCl; 1% TritonX-100, 5 mM EDTA, 10% glycerol, 10 mM dithiothreitol, and 1 × complete cocktail protease inhibitor). The cell lysates were collected and centrifuged at 12,000 rpm for 20 min, and the supernatants were incubated with 10μg of anti-FLAG or anti-hexon antibodies and 50 μL of a 50% slurry of protein A/G plus agarose at 4 °C for 6 h. Beads were washed six times with the lysis buffer and boiled with 2 × SDS loading buffer for 10 min. Samples were subjected to 12% SDS-PAGE gel electrophoresis and stained with coomassie brilliant blue R-250 or silver staining. After separation of proteins on SDS-PAGE gel, the bands of interest were sliced and analyzed by liquid chromatography-mass spectrography (LC-MS). The scores of identified proteins were computed by Mascot in the database of Uniprot (available online: https://www.uniprot.org/taxonomy/9031). The identified FAdV-4 hexon-binding protein candidates were mapped to different biological pathways according to functional categories of the Kyoto encyclopedia of genes and genomes (KEGG) pathway database (available online: http://www.genome.ad.jp/kegg/pathway.html).

### 2.5. Western Blot Analyses

The cell lysates were prepared as described above, and the samples were boiled with 1×SDS loading buffer for 10 min. Equal amounts of protein were separated by SDS-PAGE and transferred onto polyvinylidene difluoride (PVDF) membrane. After blocking with 5% skimmed milk, the membranes were incubated with the indicated antibodies. Blots were developed using an ECL kit.

### 2.6. Immunoprecipitation

The immunoprecipitation approach used to analyze protein interaction has been previously described [31]. Briefly, for the immunoprecipitation of Myc-CCT7 with Flag-hexon, LMH cells or HeLa cells were cotransfected with PRK5-flag-hexon, pCMV-Myc-cct7 or empty vectors as controls. Twenty-four hours after transfection, cell lysates were prepared using lysis buffer. The cell lysates were subjected to immunoprecipitation with anti-FLAG antibody at 4 °C for 3 h and then mixed with 20 μL of a 50% slurry of protein A/G plus-agarose and incubated for another 3 h. Beads were washed six times with lysis buffer and boiled with 2 × SDS loading buffer for 10 min. The samples were subjected to Western Blot analyses. For the endogenous pulldown assay, LMH cells were infected with FAdV-4. Twenty-four hours after infection, the cell lysates were subjected to immunoprecipitation with anti-hexon antibody and immunoblotted with anti-hexon or anti-CCT7 antibodies. For the immunoprecipitation of truncated Myc-CCT7 (Δ1–Δ6) with Flag-hexon, truncated pCMV-Myc-cct7 (Δ1–Δ6) were individually cotransfected with PRK5-flag-hexon in LMH cells. Twenty-four hours after transfection, the cell lysates were subjected to immunoprecipitation with anti-FLAG antibody and immunoblotted with anti-FLAG or anti-Myc antibodies.

### 2.7. Transmission Electron Microscopy 

For ultrastructural analysis, LMH cells were mock infected or infected with FAdV-4 at a multiplicity of infection of 1 for 24 h. Ultra-thin sections were observed using a Hitachi HT7700 transmission electron microscope (Hitachi Ltd., Tokyo, Japan) at 100 kV.

### 2.8. Confocal Laser Scanning Microscopy Assays

LMH cells (2 × 10^5^) were seeded onto coverslips in 24-well plates and cultured overnight before transfection with pEGFP-hexon and pDsRed-cct7. Twenty-four hours after transfection, cells were fixed with 4% paraformaldehyde, and the nuclei were stained with DAPI. For the examination of viral hexon in FAdV-4-infected cells, LMH cells were mock infected or infected with FAdV-4 at an MOI of 1. Two hours after infection, cells were transfected with pDsRed-cct7. Twenty-two hours after transfection, LMH cells were fixed with 4% paraformaldehyde, permeabilized with 0.2% Triton X-100 for 15 min, blocked with 1% bovine serum albumin (BSA), and then probed with mouse anti-hexon polyclonal antibodies followed by FITC conjugated goat anti-mouse IgG antibodies. After three washes with phosphate-buffered saline (PBS), the cell nuclei were stained with DAPI. The samples were analyzed using a confocal laser scanning microscope. To determine the localizations of hexon and endogenous CCT7, LMH cells were mock infected or infected with FAdV-4 at an MOI of 1. Twenty-four hours after infection, LMH cells were fixed with 4% paraformaldehyde, permeabilized with 0.2% Triton X-100 for 15 min, blocked with 1% bovine serum albumin, and then probed with mouse anti-hexon and rabbit anti-CCT7 antibodies followed by TRITC conjugated goat anti-mouse IgG antibodies and DyLight 488 affiniPure goat anti-rabbit IgG antibodies. After three washes with PBS, the cell nuclei were stained with DAPI. The samples were analyzed by using a confocal laser scanning microscope (Olympus Corporation, Tokyo, Japan).

### 2.9. Knockdown of CCT7 by RNAi

The small interfering RNAs (siRNAs) were designed and synthesized by Genepharma Company (Suzhou, China) and used to knock down the expression of CCT7 in LMH cells. The sequences of siRNA for targeting CCT7 in LMH cells included RNAi#1 (sense: 5′- GCA AAG ACU UGC ACA AUA ATT-3′, and antisense: 5′-UUA UUG UGC AAG UCU UUG CTT-3′), RNAi#2 (sense: 5′-GGA ACA UCC UGU AUG ACA ATT-3′, and antisense: 5′-UUG UCA UAC AGG AUG UUC CTT-3′), RNAi#3 (sense: 5′-GCU GCU GAU AGG UGC UUA UTT-3′, and antisense: 5′-AUA AGC ACC UAU CAG CAG CTT-3′), and negative siRNA control (sense: 5′-UUC UCC GAA CGU GUC ACG UTT-3′, and antisense: 5′-ACG UGA CAC GUU CGG AGA ATT-3′). LMH cells were transfected with siRNA using jetPRIME reagent per the manufacturer’s instruction. Double transfections were performed at 24-h intervals. Twenty-four hours after the second transfection, cells were harvested for further analysis.

### 2.10. Examination of Hexon in CCT7 Overexpression or Knockdown Cells

LMH cells were seeded in 12-well plates and cultured twenty-four hours before being cotransfected with pRK5-flag-hexon and pCMV-Myc-cct7 or control empty vector plasmids; twenty-four hours after transfection, the cell lysates were collected and centrifuged at 12,000 rpm for 20 min, and the supernatants were examined by Western Blot using anti-FLAG, anti-Myc or anti-GAPDH antibodies. For the measurement of hexon protein in CCT7 RNAi or control RNAi cells, LMH cells were treated with CCT7 RNAi or control RNAi, followed by being transfected with pRK5-flag-hexon or infection with FAdV-4 at an MOI of 0.1. Twenty-four hours post-transfection or infection, cell lysates were prepared as above described and examined by Western Blot anti-FLAG, anti-CCT7, anti-GAPDH, or anti-FAdV-4 hexon antibodies. For protein synthesis inhibition assay with cycloheximide (CHX), LMH cells were cotransfected with pCMV-Myc-cct7 and pRK5-flag-hexon or a control empty vector. Twelve hours after transfection, cells were treated with CHX (50 μg/mL). Cell lysates were harvested at different points (0, 1, 2, 3, 4, and 5 h) and examined with Western Blot using anti-FLAG, anti-Myc or anti-GAPDH antibodies. For CCT7 knockdown assay, LMH cells were treated with CCT7 RNAi or control RNAi and transfected with pRK5-flag-hexon. Twelve hours after transfection, cells were treated with CHX (50 μg/mL). Cell lysates were harvested at different points (0, 0.5, 1.0, 1.5, 2.5, and 3.5 h) and examined with Western Blot using anti-FLAG, anti-CCT7 or anti-GAPDH antibodies. The levels of hexon expression were quantified by densitometry and normalized to that of GAPDH.

### 2.11. Measurement of FAdV-4 Growth in LMH Cells

LMH cells receiving CCT7-specific siRNA or control siRNA were infected with FAdV-4 at an MOI of 1, and cell cultures were collected at different time points (12, 24, 48, and 72 h) after infection. The viral titers in the supernatants or cell cultures were titrated using 50% tissue culture infective doses (TCID_50_) as previously described [32]. Briefly, the viral solution was 10-fold serially-diluted in Waymouth’s Medium. A 100 μL aliquot of each diluted sample was added to the well of 96-well plates, followed by the addition of 100 μL of LMH cells at a density of 3 × 10^5^ cells/ml. Cells were cultured for 5 days at 37 °C in 5% CO_2_. Tissue culture wells with a cytopathic effect were considered to be positive.

### 2.12. Statistical Analysis

The significance of the differences between CCT7 RNAi cells and controls in the levels of hexon in cells and in viral growth was determined by a Mann-Whitney test or analysis of variance (ANOVA).

## 3. Results

### 3.1. Replication of FAdV-4 HuBWH in LMH cells

**Figure 1:**
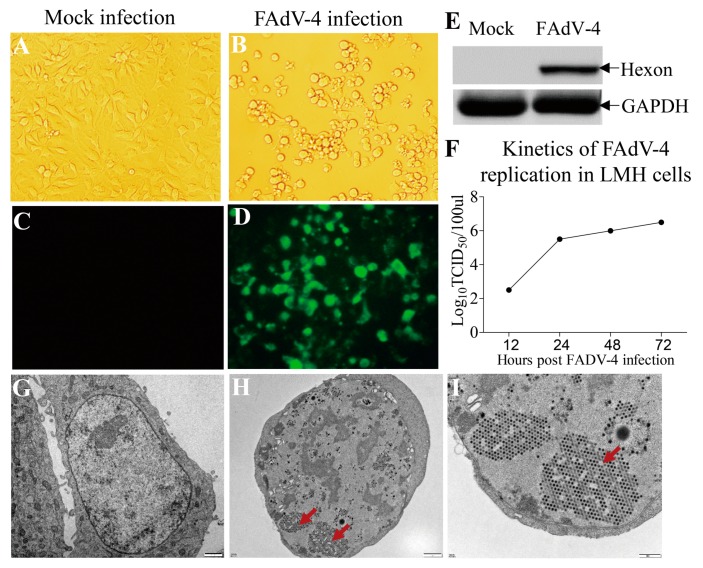
Replication of FAdV-4 strain HuBWH in LMH cells. (**A**–**D**) LMH cells were mock infected (A and C) or infected with FAdV-4 at an MOI of 1 (**B**,**D**). Twenty-four hours after FAdV-4 infection, cells were subjected to IFA staining using mouse anti-hexon polyclonal antibodies followed by FITC-conjugated goat anti-mouse IgG antibodies and were visualized under a fluorescence microscope. Magnification, ×400. (**E**) Detection of the viral protein hexon in FAdV-4-infected or mock-infected LMH cells by Western Blot using an anti-hexon McAb. (**F**) Examination of FAdV-4 replication in LMH cells. LMH cells were infected with FAdV-4 at an MOI of 1. At different time points (12, 24, 48, and 72 h) after FAdV-4 infection, the viral titers in the cell cultures were determined by TCID_50_. (G to I) Examination of FAdV-4 HuBWH particles by TEM. LMH cells were mock infected (**G**) or infected with FAdV-4 at an MOI of 1 for 24 h (**H**). A higher-magnification view of panel H is demonstrated by (**I**) (The FAdV-4 particles are indicated by red arrows). Scale bar = 1 μm in G&H; Scale bar = 500 nm in I.

Some FAdVs are capable of replicating in multiple types of cells, including chicken embryo liver cell [33], chicken kidney cell [34] and leghorn male hepatocellular (LMH) cell [35]. The fact that the main target organ of FAdV-4 infection is chicken liver prompted us to investigate whether the FAdV-4 HuBWH strain could replicate in the LMH cell. We infected the LMH cells with the virus at an MOI of 1 and examined the viral growth with immunofluorescence antibody assay (IFA). Twenty-four hours after FAdV-4 HuBWH infection, the infected cells showed obvious cytopathology effects (CPE) (Figure 1A,B) and a large number of immunofluorescent cells could be detected after IFA staining using mouse anti-hexon polyclonal antibodies (Figure 1C,D). In addition, the hexon protein of FAdV-4 could be detected in FAdV-4-infected cells with Western Blot analysis (Figure 1E), and the virus grew very well in FAdV-4-infected LMH cells as examined by TCID_50_ (Figure 1F). Furthermore, the viral particles of FAdV-4 could be observed in LMH cells post FAdV-4 infection, showing a well-arranged crystal lattice structure in LMH cells as observed under a transmission electron microscope (Figure 1G–I). These results clearly indicate that FAdV-4 HuBWH can replicate in LMH cells.

### 3.2. Screening and Identification of FAdV-4 Hexon Interacting Cellular Proteins in LMH Cells

**Figure 2:**
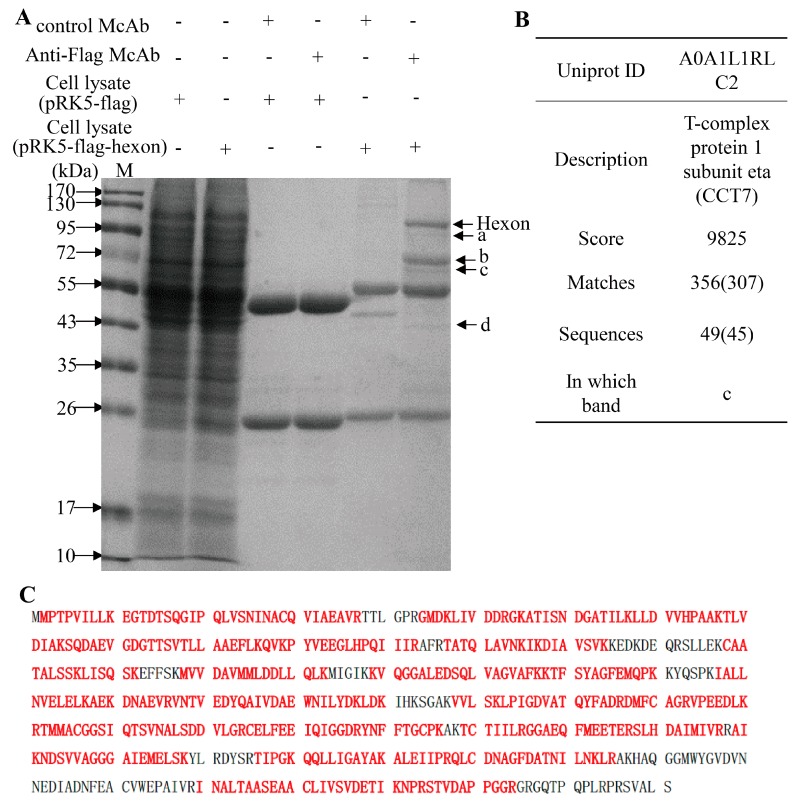
Screening for hexon-interacting proteins in LMH cells. (**A**–**C**) LMH cells transfected with pRK5-flag-hexon or pRK5-flag and subjected to a pull-down assay (**A**). The arrow-pointed protein bands in (**A**) were further analyzed by liquid chromatography-mass spectrograph (LC-MS), and the cellular protein CCT7 was identified from the arrow-pointed protein band c (**B**). The alignment of the chicken CCT7 sequences (UniProtKB: A0A1L1RLC2) with its matched peptides is shown in bold red as identified by LC-MS (**C**).

The capsid of FAdVs is composed of 720 hexon subunits arranged as 240 trimers and 12 vertex penton capsomers with one or two fibers jutting from the surface [10]. To investigate whether FAdV-4 hexon interacts with cellular proteins of the host, we transfected LMH cells with pRK5-FLAG-hexon and performed a pull-down assay using anti-FLAG monoclonal antibody (McAb). We found that there were four extra clear protein bands in the immunoprecipitates of the mixture of anti-FLAG McAb with pRK5-FLAG-hexon transfected cell lysate as compared to that of controls as demonstrated by SDS-PAGE (Figure 2A), indicating that some cellular proteins interacted with hexon. To analyze the amino acid sequence of these candidate proteins interacting with hexon, we cut-down the protein bands of interest and performed a mass spectrometry. As a result, the arrow-pointed bands in Figure 2A contained 1084 proteins. Among these proteins, CCT7 has been identified with a high score of 9825 and its sequence coverage was 80% (Figure 2B,C). Furthermore, CCT7 could also be pulled-down by anti-hexon McAb from the lysates of FAdV-4 infected cells (Figure S1A–C). Thus, CCT7 aroused our interest for further investigation.

### 3.3. Hexon Interacts with the Cellular Protein CCT7

**Figure 3:**
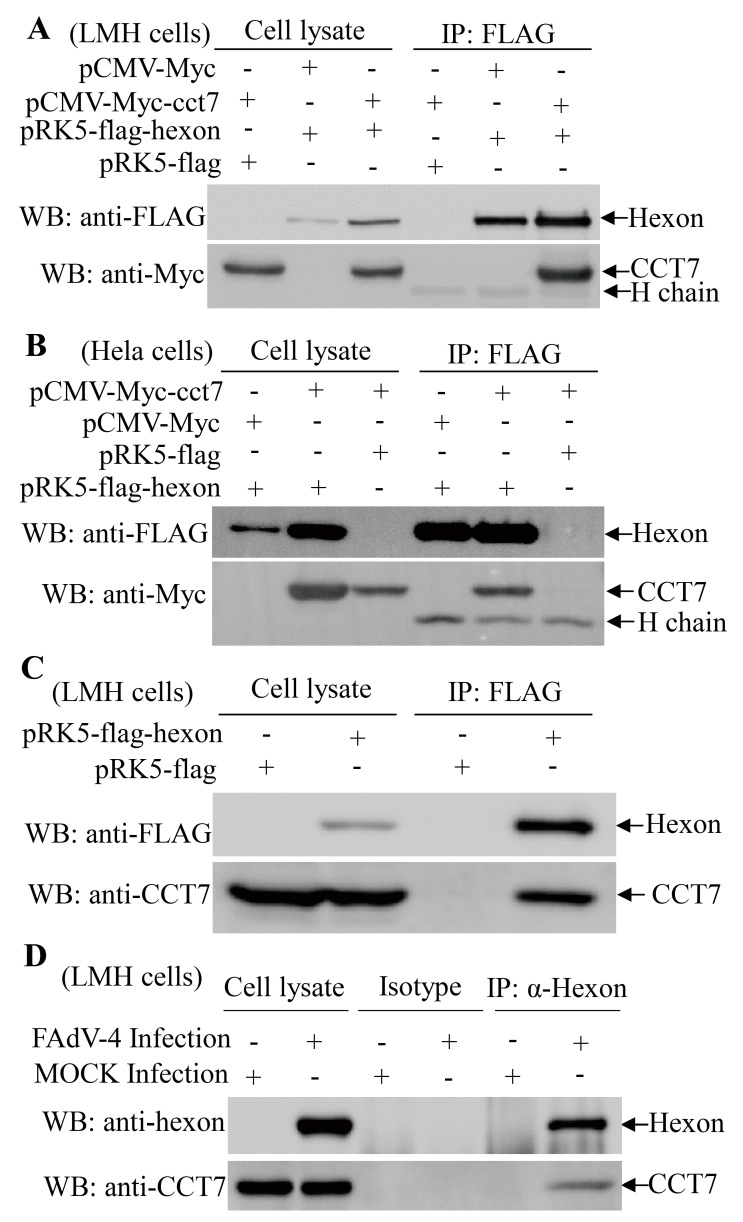
Interaction of FAdV-4 hexon with cellular protein CCT7. (**A**,**B**) The interaction of hexon with exogenous CCT7. LMH cells (**A**) and HeLa cells (**B**) were transfected with the indicated expression plasmids and immunoprecipitated (IP) with anti-FLAG antibodies. Hexon and CCT7 in the immune complex were examined with Western Blot using anti-FLAG and anti-Myc McAbs, respectively in the cell lysates and in the immunoprecipitates too. (**C**) The interaction of hexon with endogenous CCT7 in LMH cells. LMH cells were transfected with pRK5-FLAG-hexon or empty vector as control. Twenty-four hours after transfection, cell lysates were prepared and immunoprecipitated with anti-FLAG antibody. Both the cell lysates and the immunoprecipitates were immunoblotted with anti-FLAG or anti-CCT7 antibodies. (**D**) Interaction of hexon with endogenous CCT7 in FAdV-4-infected cells. LMH cells were mock infected or infected with FAdV-4 at an MOI of 1, and immunoprecipitation assays were performed with an anti-hexon McAb. CCT7 in the immune complex was examined with Western Blot using anti-CCT7 polyclonal antibodies. The “H chain” means the heavy chain of the antibody. The “isotype” means isotype (IgG2b) control antibody of anti-FAdV-4 hexon monoclonal antibody.

Since CCT7 showed up as a potential cellular protein interacting with hexon, we set out to determine the interaction of hexon with CCT7 in cells. We constructed a plasmid that allows the expression of Myc-CCT7 for analyzing its interaction with hexon in LMH cells. When the lysates of cells expressing both Flag-hexon and Myc-CCT7 were immunoprecipitated with anti-FLAG antibody, Myc-CCT7 was detected in the precipitate, indicating that hexon interacted with ectopically-expressed CCT7 in chicken cells (Figure 3A). A similar result was obtained in an experiment using the HeLa cells (Figure 3B), indicating that the interaction observed between these two proteins is not cell type-specific. Furthermore, we transfected LMH cells with pRK5-FLAG-hexon plasmids and performed an immunoprecipitation assay with anti-FLAG McAb. The binding of FLAG-hexon with endogenous CCT7 was readily detectable in cells expressing the protein hexon (Figure 3C). To further substantiate the binding of hexon to CCT7, we infected LMH cells with FAdV-4 HuBWH and examined the interaction of hexon with endogenous CCT7 using pulldown assays. Consistently, the endogenous CCT7 was also detected in FAdV-4-infected cells but not in mock-infected controls (Figure 3D). These results clearly demonstrate that viral capsid protein hexon interacts with CCT7 in cells.

### 3.4. Hexon Colocalizes with CCT7 in Cells

**Figure 4:**
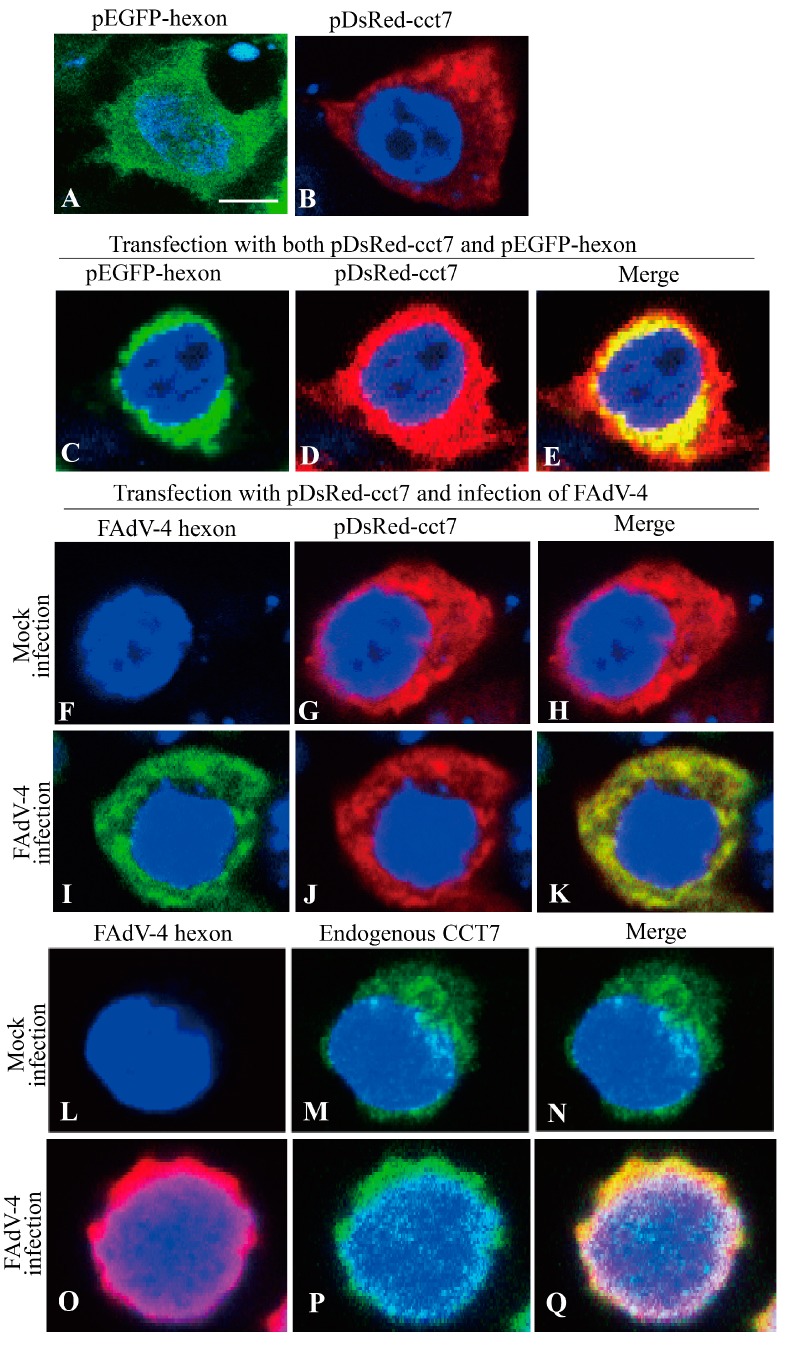
Colocalization of hexon with CCT7 in LMH cells. (**A**–**E**) Localization of hexon and exogenous CCT7 in LMH cells. LMH cells were seeded onto 24-well plates with coverslips in the wells and cultured overnight. Cells were transfected with pEGFP-hexon (**A**), pDsRed-cct7 (**b**) or both pEGFP-hexon and pDsRed-cct7 (**C**–**E**). Twenty-four hours after infection, cells were fixed and the cell nuclei were counterstained with DAPI (blue). The cell samples were observed with a confocal laser scanning microscope. (**F**–**K**) Colocalization of FAdV-4 hexon with exogenous CCT7 in FAdV-4-infected cells. LMH cells were mock infected or infected with FAdV-4 at an MOI of 1. Two hours after infection, cells were transfected with the indicated expression plasmids. Twenty-two hours after transfection, cells were fixed and probed with mouse anti-hexon polyclonal antibodies, followed by incubation with FITC-conjugated goat anti-mouse antibodies (green). Nuclei were counterstained with DAPI (blue). (**L**–**Q**) Colocalization of FAdV-4 hexon with endogenous CCT7 in FAdV-4-infected cells. LMH cells were mock infected or infected with FAdV-4 at an MOI of 1. Twenty-four hours after infection, cells were fixed and probed with mouse anti-hexon and rabbit anti-CCT7 polyclonal antibodies, followed by incubation with TRITC-conjugated goat anti-mouse antibodies (red) and DyLight 488 affiniPure goat anti-rabbit IgG antibodies (green). Nuclei were counterstained with DAPI (blue). The cell samples were observed with a confocal laser scanning microscope. Scale bar = 5 μm.

To determine the subcellular localizations of hexon and CCT7, we performed confocal microscopy assays with LMH cells transfected to express DsRed-CCT7 and GFP-hexon. Transfection of LMH cells with DsRed-CCT7 and GFP-hexon indicated that both CCT7 and hexon were primarily located in the cytoplasm (Figure 4A,B). When LMH cells were transfected with these two plasmids together, we observed colocalization of hexon and CCT7 in the cytoplasm of transfected cells (Figure 4C–E), supporting the above findings that CCT7 interacted with hexon in cells. To further consolidate these results, we examined the colocalization of CCT7 with hexon in FAdV-4-infected LMH cells. We transfected LMH cells with pDsRed-cct7 and infected these cells with FAdV-4 at an MOI of 1 and performed immunofluorescent antibody assay (IFA) using anti-hexon antibodies. As shown in Figure 4F-K, CCT7 was also colocalized with hexon in the cytoplasm of FAdV-4-infected cells. To determine whether endogenous CCT7 colocalizes with hexon in FAdV-4-infected cells, we performed IFA using mouse anti-hexon and rabbit anti-CCT7 antibodies in FAdV-4-infected LMH cells. As expected, endogenous CCT7 was colocalized with hexon in FAdV-4-infected cells (Figure 4L–Q). These results strongly support the finding that FAdV-4 hexon interacts with CCT7 in cells.

### 3.5. The Amino Acid Residues 137 to 272 of CCT7 is Responsible for Interaction with the Hexon

**Figure 5:**
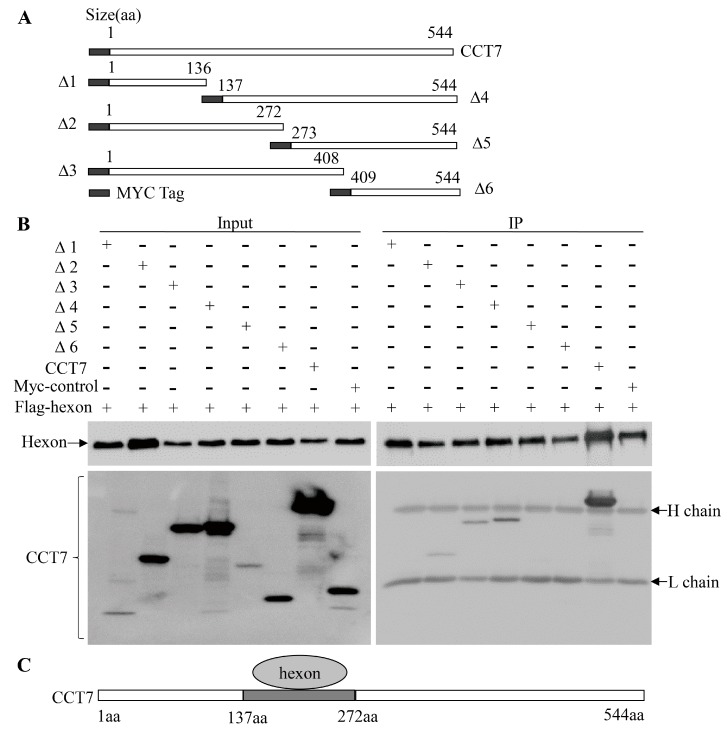
The portion of CCT7 from amino acids 137–272 is responsible for binding to hexon. (**A**) Schematics represent the genes encoding the full-length CCT7 and truncated CCT7 molecules (from Δ1 to Δ6). (**B**) Interaction of Flag-hexon with different truncated CCT7 mutants. LMH cells were transfected with full-length pCMV-myc-cct7, the indicated truncated mutants or pCMV-myc-control (encode a 17kDa control protein). Cell lysates were prepared and immunoprecipitated with anti-Flag McAb. The pellets were examined with Western Blot using anti-Myc and anti-FLAG antibodies. (**C**) Schematic representing the domain (137–272aa) of CCT7 responsible for binding to hexon. The “H chain” means the heavy chain of antibody and the “L chain” means the light chain of antibody.

To determine the region of CCT7 responsible for interacting with hexon, we constructed a series of CCT7 deletion mutants fused to a MYC tag (Figure 5A). These CCT7 derivatives were individually expressed in LMH cells and their ability to interact with hexon was examined by immunoprecipitation. Our results show that, with the exception of mutants (Δ1, Δ5 and Δ6) lacking residues 137–272, other CCT7 mutants including the residues 137–272 (Δ2, Δ3 and Δ4) retained the ability to interact with hexon (Figure 5B), indicating that this region of CCT7 is important for interaction with hexon (Figure 5C).

### 3.6. CCT7 is Required for Maintaining the Stability of Hexon in Cells

**Figure 6:**
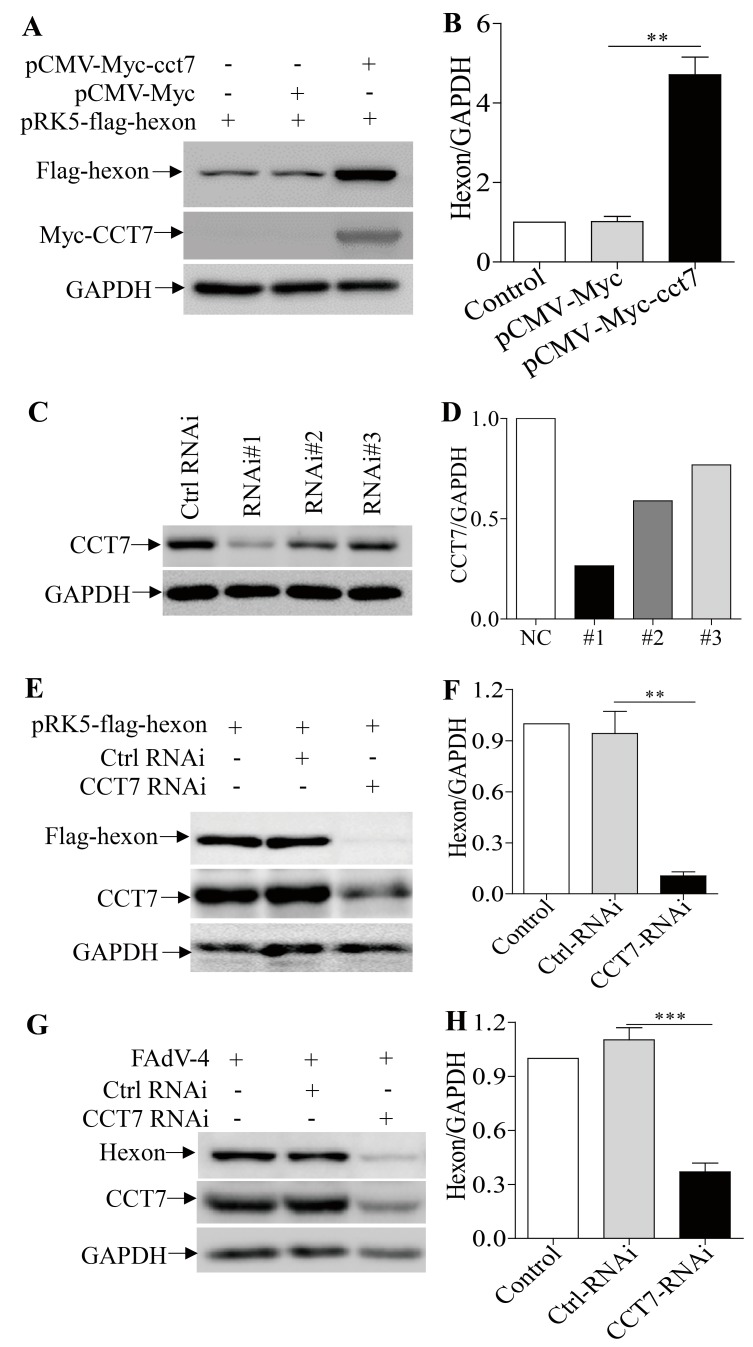
CCT7 is required for the presence of hexon. (**A**,**B**) Overexpression of CCT7 enhanced hexon levels in cells transfected with pRK5-flag-hexon. LMH cells were transfected with the indicated plasmids; twenty-four hours after transfection, the cell lysates were prepared and examined by Western Blot (**A**). The levels of hexon in (**A**) were quantified by densitometry and normalized to that of GAPDH (**B**). The level of hexon in controls was set at 1.0. (**C**,**D**) Effects of CCT7 RNAi on the expression of endogenous CCT7. LMH cells were transfected with siRNA (RNAi#1-3) or controls. Twenty-four hours after the second transfection, cell lysates were prepared and examined by Western Blot using anti-CCT7 antibodies (**C**). Endogenous GAPDH was used as an internal control. The density of bands in (**C**) was quantitated by densitometry as above described (**D**). (**E**,**F**) knockdown of CCT7 reduced hexon expression in cells. LMH cells were treated with CCT7 RNAi or control RNAi and transfected with pRK5-flag-hexon (**E**). Twenty-four hours post-transfection, cell lysates were prepared, and the expression levels of hexon were examined by Western Blot using anti-hexon McAb. The levels of hexon expression in (**E**) were quantified by densitometry and normalized to that of GAPDH (**F**). The expression levels of hexon in normal control LMH cells was set at 1.0. (**G**,**H**) LMH cells were treated with CCT7 RNAi or control RNAi, followed by infection with FAdV-4 at an MOI of 0.1. Twenty-four hours post-infection, cell lysates were prepared, and the expression of hexon was examined by Western Blot using anti-hexon McAb (**G**). The levels of hexon expression in (**G**) were quantified by densitometry and normalized to that of GAPDH (**H**). The expression level of hexon in normal control LMH cells was set at 1.0. Data are represented as mean ± SD, *n* = 3. ** stands for *p* < 0.01 and *** for *p* < 0.001.

The binding of hexon to CCT7 suggests that CCT7 is important to FAdV-4 hexon in cells. To determine the requirement of CCT7 for hexon in cells, we transfected cells with pRK5-flag-hexon along with pCMV-Myc-cct7, and examined the expression of hexon in cells. As a result, overexpression of CCT7 markedly enhanced the content of hexon in pRK5-flag-hexon-transfected cells compared to that in control cells (Figure 6A,B). On the contrary, knockdown of endogenous CCT7 by RNAi significantly reduced hexon levels in pRK5-flag-hexon-transfected cells compared to that of the RNAi control (Figure 6C–F), so did the knockdown of CCT7 in FAdV-4-infected cells (Figure 6G,H). These results clearly show that CCT7 is required for the presence of hexon in cells.

**Figure 7:**
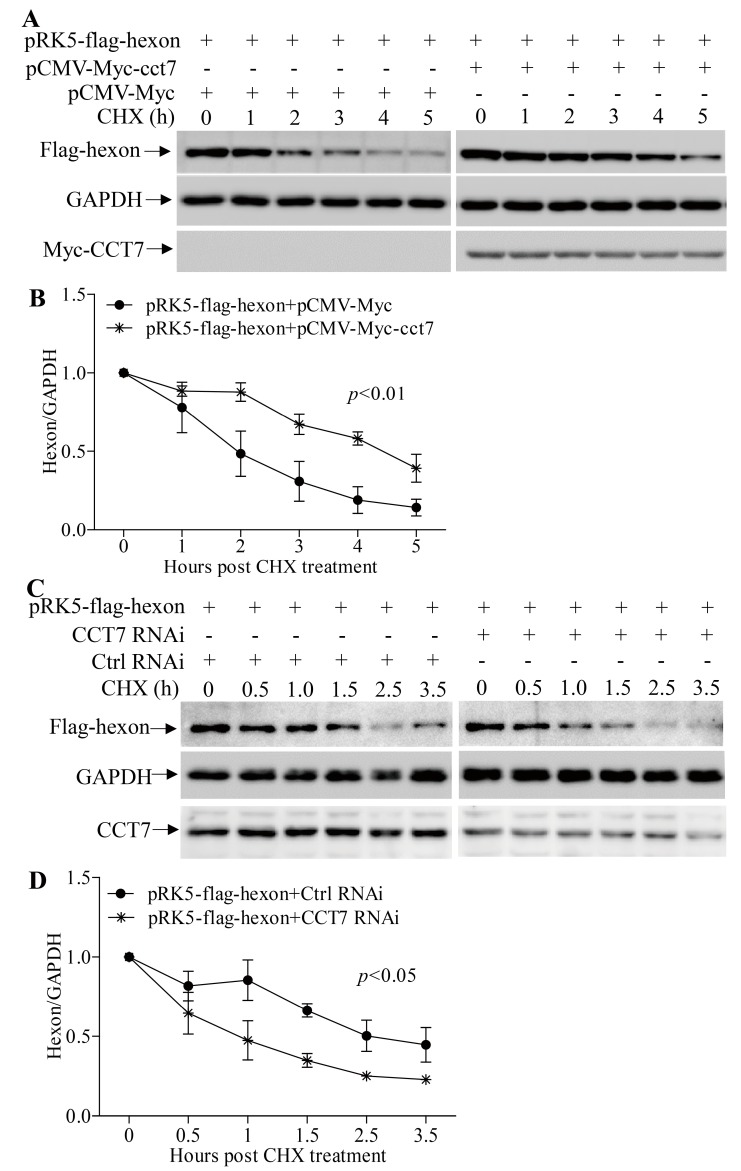
Involvement of CCT7 in hexon reduction in LMH cells. (**A**,**B**) LMH cells were cotransfected with pCMV-Myc-cct7 and pRK5-flag-hexon or control empty vector. Twelve hours after transfection, cells were treated with cycloheximide (CHX, 50 μg/mL) at different time points (0, 1, 2, 3, 4, and 5 h) before being harvested. Cell lysates were prepared and examined with Western Blot using the indicated antibodies. The levels of Flag-hexon in (**A**) were quantified by densitometry and normalized to that of GAPDH (**B**). The value of normalized Flag-hexon at time 0 h was set at 1.0. (**C**,**D**) LMH cells were treated with CCT7 RNAi or control RNAi and transfected with pRK5-flag-hexon. Twelve hours after transfection, cells were treated with cycloheximide (CHX, 50 μg/mL) at different time points (0, 0.5, 1.0, 1.5, 2.5, and 3.5 h) before being harvested. Cell lysates were prepared and examined with Western Blot using indicated antibodies. The levels of Flag-hexon in (**C**) were quantified by densitometry and normalized to that of GAPDH (**D**). The value of normalized Flag-hexon at time point 0 h was set at 1.0. The significance of the difference was determined by ANOVA.

Since TRiC/CCT plays a key role in the cellular stability of its client proteins [36,37] and CCT7 is required for the presence of hexon in cells, it would be intriguing to examine the effect of CCT7 on the stability of hexon. We transfected LMH cells with pCMV-Myc-cct7 and pRK5-flag-hexon, treated cells with cycloheximide (CHX), an inhibitor of protein synthesis, and examined the contents of hexon in cells at different time points (0, 1, 2, 3, 4, and 5 h) post CHX treatment. Our results show that the contents of hexon quickly reduced in cells post CHX treatment (Figure 7A,B). In contrast, this reduction could be significantly mitigated in cells with overexpression of CCT7. On the contrary, knockdown of CCT7 facilitated the reduction of hexon in pRK5-flag-hexon-transfected cells (Figure 7C,D). These data indicate that CCT7 is involved in maintaining the stability of hexon in cells.

### 3.7. CCT7 Facilitates FAdV-4 Replication in Cells

**Figure 8:**
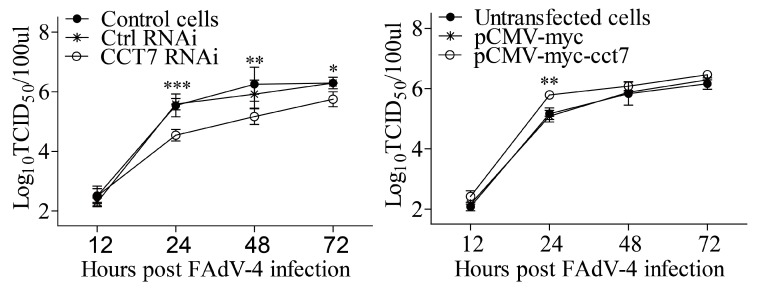
Effects of CCT7 on FAdV-4 growth in LMH cells. (**A**) Knockdown of CCT7 suppresses FAdV-4 growth. LMH cells were treated with RNAi to knockdown endogenous CCT7 expression as described above, followed by infection with FAdV-4 at an MOI of 1. At different time points (12, 24, 48, and 72 h) post FAdV-4 infection, the viral titers in the cell cultures were determined by TCID50 analysis using 96-well plates. The significance of the difference between CCT7 RNAi treatment and RNAi controls was performed by ANOVA. (**B**) LMH cells were transfected with pCMV-myc-cct7 or empty vectors, followed by infection with FAdV-4 at an MOI of 1. At different time points (12, 24, 48, and 72 h) post FAdV-4 infection, the viral loads in the cell cultures were determined by TCID_50_ using 96-well plates. The significance of the difference between pCMV-myc-cct7 transfected cells and controls in viral growth was determined by ANOVA. * stands for *p* < 0.05, ** stands for *p* < 0.01 and *** stands for *p* < 0.001.

As the CCT7 helps to stabilize FAdV-4 hexon, a key viral capsid protein, CCT7 should therefore affect FAdV-4 replication. To test this hypothesis, we examined viral replication in cells with CCT7 knockdown or overexpression at different time points post FAdV-4 infection. As a result, knockdown of CCT7 by RNAi markedly inhibited FAdV-4 replication in cell cultures compared to that in controls (Figure 8A). On the contrary, overexpression of CCT7 in LMH cells enhanced viral loads in the cell culture compared with that of controls (Figure 8B). These data indicate that CCT7 is required for FAdV-4 replication in cells.

## 4. Discussion

FAdV-4 is a hepatotropic virus that causes inclusion body hepatitis and hydropericardium syndrome. It has been reported that FAdV-4 induces liver injury via apoptosis, autophagy, and a severe inflammatory response [29]. Among the organs of FAdV-4 infected chickens, the liver had the highest viral load, followed by the spleen, and a great number of basophilic inclusion bodies were observed in the liver [2,30]. Although hydropericardium syndrome was found in FAdV-4-infected chickens, there was no noted pathological changes in the heart [30] and FAdV-4 infection did not induce apoptosis in cardiomyocytes [38]. 

In the present study, we found that FAdV-4 replicated well and caused significant cytopathic effects in LMH cells (Figure 1). Using pull-down assay and mass spectrometry, we identified multiple potential proteins that interact with hexon (Figure 2). Among these cellular proteins, T-complex polypeptide 1 subunit eta (CCT7) attracted our attention because T-complex protein-1 (TCP-1) ring complex (TRiC) has been reported to regulate the replication of several viruses. Six (CCT1-CCT6) out of the eight subunits of the CCT complex were identified as Dengue virus (DENV) non-structural (NS) protein partners and components of CCT complex are required for DENV replication [39]. TRiC participated in hepatitis C virus replication and virion production possibly through an interaction between CCT5 and NS5B [24]. As a key factor in viral replication, murine cytomegalovirus (MCMV) M72 interacted with, and was a substrate of, the TRiC complex [25]. Thus, we assumed that CCT7 might be involved in FAdV-4 replication in cells via interaction with hexon, a FAdV-4 capsid protein. Interestingly, we found that the knockdown of CCT7 significantly reduced hexon levels in LMH cells (Figure 6E–H). On the contrary, overexpression of CCT7 considerably enhanced hexon levels (Figure 6A,B). Furthermore, we found that CCT7 was involved in maintaining the stability of hexon in cells (Figure 7), and CCT7 is required for the replication of FAdV-4 (Figure 8). Clearly, our results show a direct and functional interaction of FAdV-4 hexon with the cellular protein CCT7.

TRiC has been shown to be involved in the folding and viral assembly of several viral capsid proteins such as retroviral Gag protein [40], hepatitis B virus core protein [41] and reovirus σ3 outer-capsid protein [28]. Our results also show that TRiC subunit CCT7 is related to hexon levels in LMH cells. Although CCT7 has an effect on the half-life of hexon, it cannot be ruled out that TRiC may be involved in the folding of hexon protein or plays a role in the assembly of FAdV-4 capsid proteins. It was found that purified CCT4 or CCT5 subunits from *E. coli* are capable of forming homo-oligomeric chaperonin-like complexes independent of the other seven subunits [42]; these novel complexes are not only morphologically similar to TRiC, but also have the function of folding substrate proteins. In the present study, overexpression of CCT7 alone can increase the amount of hexon protein in cells with pRK5-flag-hexon transfection, suggesting that chicken CCT7 is likely to form homo-oligomeric chaperonin-like complex as well as human CCT4 and CCT5 without relying on the other seven subunits.

As a small molecule activator of heat shock transcription factor 1 (HSF1), HSF1A has been proven to inhibit TRiC activity via binding to TRiC subunits in vivo and in vitro [43]. It has been reported that CCT4/5 directly interacted with the *Clostridium difficile* toxins A (TcdA) and B (TcdB). Suppression of CCT5 function by compound HSF1A blocked the cytotoxic effects of TcdA and TcdB [44]. Based on these, we propose that HSF1A may also have a negative impact on FAdV-4 replication. More efforts will be required to determine whether the application of compound HSF1A to flocks can reduce the mortality of chickens with FAdV-4 infection.

In the present study, FAdV-4 infection of LMH cells caused severe cytopathic effects, which might result from FAdV-4-induced apoptosis, because we noted that hexon potentially interacted with some proteins of the apoptotic pathway based on our pull-down assays and KEGG analysis (Figure S2 and Table S3), such as eukaryotic translation initiation factor 2 subunit 1 (eIF2α) and cathepsin D. eIF2α plays a critical role in endoplasmic reticulum stress-induced apoptosis [45,46], and Cathepsin D can trigger bax activation and promote apoptosis [47,48]. It has been reported that vacuolar protein sorting 52 (VPS52) activated the apoptotic pathway via cathepsin D [49]. In addition, hexon reduced quite quickly in cells post-treatment with CHX, an inhibitor for protein synthesis, and the results from the KEGG analysis indicate that hexon protein is related to the proteasome degradation pathway (Figure S2 and Table S1), suggesting that hexon may be degraded by the proteasome degradation pathway. More efforts will be required to determine whether FAdV-4 hexon is degraded in cells via the ubiquitin-mediated proteasome pathway and if CCT7 is involved in this process. 

In conclusion, our data show that CCT7 interacted with hexon in FAdV-4-infected cells. Knockdown of CCT7 by RNAi reduced hexon in LMH cells associated with the inhibition of FAdV-4 growth, while overexpression of CCT7 significantly mitigated the reduction of hexon and facilitated FAdV-4 growth. Thus, CCT7 is required for the replication of FAdV-4 in LMH cells via interaction with viral hexon protein.

## Supplementary Materials

Supplementary materials are available online at http://www.mdpi.com/1999-4915/11/2/107/s1. Figure S1. Screening and identification of FAdV-4 hexon-interacting cellular proteins in FAdV-4-infected LMH cells; Figure S2. KEGG (Kyoto Encyclopedia of Genes and Genomes) pathway enrichment analyses of the protein samples in Figure 2A shows the identified FAdV-4 hexon-binding protein candidates. Tables S1–S3. Statistics of KEGG enrichment in Supplementary Figure 2.
