# Supplementary material for: Requirement of Cellular Protein CCT7 for the Replication of Fowl Adenovirus Serotype 4 (FAdV-4) in Leghorn Male Hepatocellular Cells Via Interaction with the Viral Hexon Protein
Source: Viruses. 2019 Jan 27;11(2):107. doi: 10.3390/v11020107 (PMC6410038; doi:10.3390/v11020107)
Supplement: Supplementary file 1 [file viruses-11-00107-s001.zip › Supplementary Figures.pdf]

Supplementary Figure 1

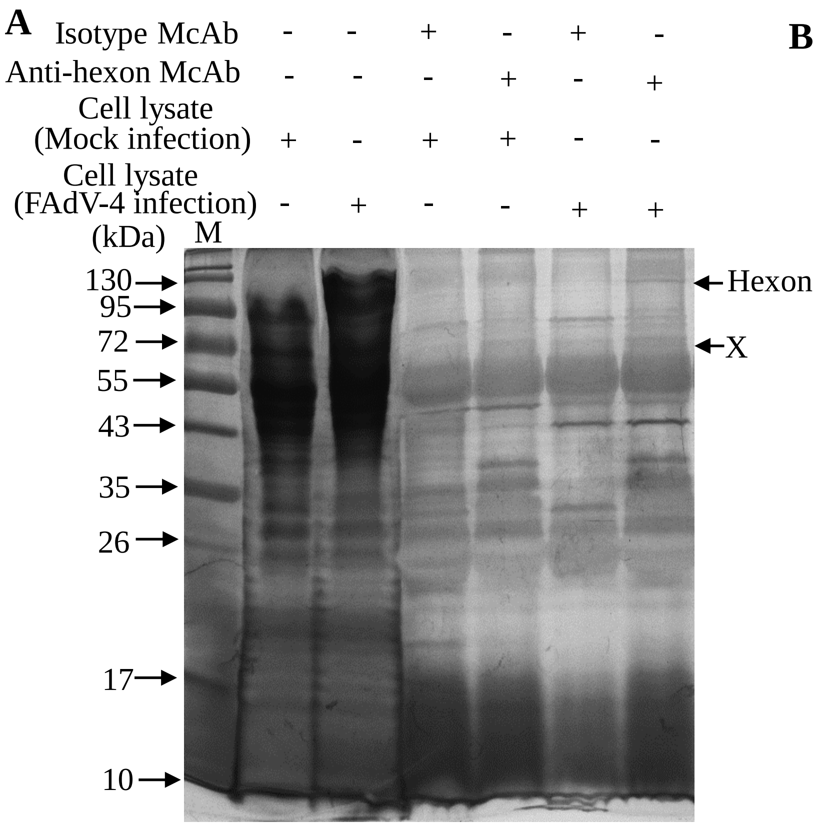

B

Uniprot ID	F1NK38
Description	T-complex protein 1 subunit eta (CCT7)
Score	34.42
Coverage	13.97
Unique Peptides	8
PSMs	20
In which band	X

C

MMPTPVILLK EGTDTSQGIP QLVSNINACQ VIAEAVRTTL GPR**GMDKLIV DDRGKATISN DGATILK**LLD VVHPAAKTLV
DIAKSQDAEV GDGTTSVTLL AAEFLKQVKP YVEEGLHPQI IIRAFR**TATQ LAVNKIKDIA VSVK**KEDKDE QRSLLEKCAA
TALSSKLISQ SKEFFSKMVV DAVMMLDDLL QLKMIGIKKV QGGALEDSQL VAGVAFKK**TF SYAGFEMQPK** KYQSPKIALL
NVELELKAEK DNAEVRVNTV EDYQAIVDAE WNILYDKLDK IHKSGAKVVL SKLPIGDVAT QYFADRDMFC AGRVPEEDLK
RTMMACGGSI QTSVNALSDD VLGRCELFEE IQIGGDRYNF FTGCPKAKTC TIILR**GGAEQ FMEETER**SLH DAIMIVRRAI
KNDSVVAGGG AIEMELSKYL RDYSRTIPGK **QQLLIGAYAK** ALEIIPRQLC DNAGFDATNI LNKLRAKHAQ GGMWYGVDVN
NEDIADNFEA CVWEPAIVRI NALTAASEAA CLIVSVDETI KNPRSTVDAP PGGRGRGRGR PHNH

Fig. S1. Screening and identification of FAdV-4 hexon-interacting cellular proteins in FAdV-4-infected LMH cells. (A-C) LMH cells were mock infected or infected with FAdV-4, and subjected to a pull-down assay. The arrow pointed protein bands in (A) were further analyzed by liquid chromatography-mass spectrography (LC-MS) (B) and the celluar protein CCT7 was identified from the arrow-pointed protein band X in (A). The alignment of the chCCT7 sequences (UniProtKB: F1NK38) with its matched peptides is shown in bold red identified by LC-MS (C).

Supplementary Fig 2

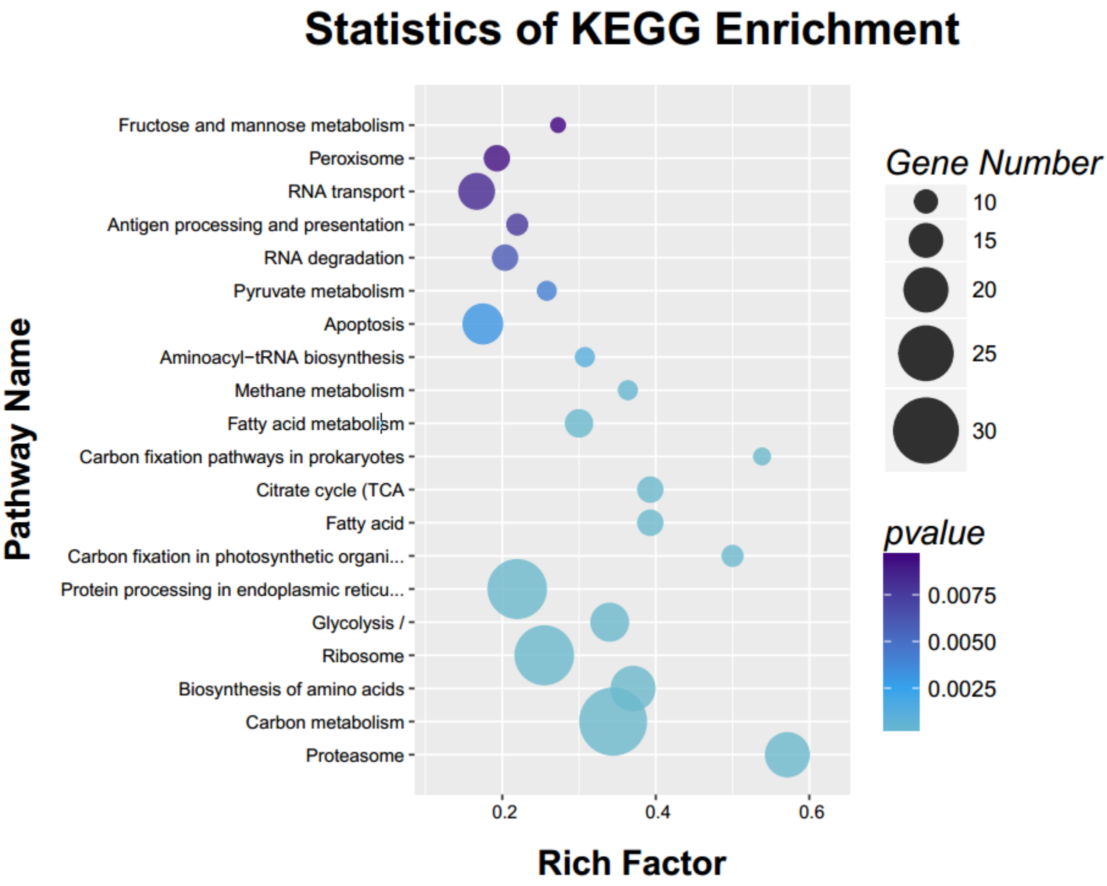

Fig. S2. KEGG (Kyoto Encyclopedia of Genes and Genomes) pathway enrichment analyses of the protein samples in Figure 2A shows the identified FAdV-4 hexon-binding protein candidates.
