# Supplementary material for: Requirement of Cellular Protein CCT7 for the Replication of Fowl Adenovirus Serotype 4 (FAdV-4) in Leghorn Male Hepatocellular Cells Via Interaction with the Viral Hexon Protein
Source: Viruses. 2019 Jan 27;11(2):107. doi: 10.3390/v11020107 (PMC6410038; doi:10.3390/v11020107)
Supplement: Supplementary file 1 [file viruses-11-00107-s001.zip › Supplementary Tables.pdf]

Table S1. Statistics of KEGG enrichment in supplementary figure 2 (a)

Pathway ID	Pathway name	Protein name (Uniprot ID)
ko03050	Proteasome	Proteasome subunit alpha type (F1NEQ6); Proteasome 26S subunit, ATPase 3 (Q5ZIT7); 26S proteasome non-ATPase regulatory subunit 13 (P84169); PSMC5 (F1NU79); 26S proteasome non-ATPase regulatory subunit 1 (Q5F418); Proteasome subunit beta (Q7ZT63); 26S proteasome non-ATPase regulatory subunit 1 (A0A1D5PI39); 26S proteasome non-ATPase regulatory subunit 2 (Q5ZLU4); Proteasome 26S subunit, ATPase 2 (Q5ZMB8); Proteasome 26S subunit, non-ATPase 11 (F1NPA2); Proteasome subunit alpha type (Q5ZJX9); Proteasome subunit alpha type (F1NC02); Proteasome subunit alpha type (A0A1D5PHL0); Proteasome subunit beta (E1BYW9); Proteasome 26S subunit, non-ATPase 6 (F1N903); Proteasome 26S subunit, ATPase 6 (Q5ZKX2); PSMD4 (R4GLK3); Proteasome subunit beta (Q6JLB2); Proteasome subunit alpha type (Q5ZLI2); RCJMB04_31h10 (Q5ZI14).
ko01200	Carbon metabolism	Isocitrate dehydrogenase [NADP] (Q5ZL82); ATP-dependent 6-phosphofructokinase (A0A1D5PG85); Transketolase (F1P1A5); Isocitrate dehydrogenase [NADP] (F1NPG2); Phosphoglycerate kinase (P51903); Succinate dehydrogenase flavoprotein subunit (F1NPJ4); Fructose-bisphosphate aldolase B (P07341); ACAT2 (F1NT20); Phosphoglycerate mutase 1 (Q5ZLN1); Fructose-bisphosphate aldolase (R4GM10); Glyceraldehyde-3-phosphate dehydrogenase (F1NH87); Malate dehydrogenase (E1BVT3); Aconitate hydratase, mitochondrial (Q8AYI3); Pyruvate carboxylase (Q8JHF6); Succinate dehydrogenase iron-sulfur subunit (A0A1D5PIF0); Glucose-6-phosphate isomerase (Q5ZMU3); HADHA (F1NI29); Propionyl-CoA carboxylase subunit alpha (F1P0M2); ATP-dependent 6-phosphofructokinase (Q90YA3); Enoyl-CoA hydratase and 3-hydroxyacyl CoA dehydrogenase (E1C1T9); Triosephosphate isomerase (P00940); Ransferase (P00508); Phosphoglycerate mutase (F1NHM9); Phosphoserine aminotransferase (F1NTK1); ACAT1 (E1C0Q5); Pyruvate kinase PKM (P00548); Alpha-enolase (P51913); Acetyltransferase component of pyruvate dehydrogenase complex (E1C6N5).
ko01230	Biosynthesis of amino acids	ATP-dependent 6-phosphofructokinase (Q90YA3); Transketolase (F1P1A5); Phosphoglycerate kinase (P51903); Pyruvate kinase PKM (P00548); Glyceraldehyde-3-phosphate dehydrogenase (P00356); Phosphoglycerate mutase 1 (Q5ZLN1); Aspartate aminotransferase (P00508); Fructose-bisphosphate aldolase B (P07341); Cystathionine gamma-lyase (E1BYF1); Aconitate hydratase (Q8AYI3); Pyruvate carboxylase (Q8JHF6); Triosephosphate isomerase (P00940); Isocitrate dehydrogenase (Q5ZL82); Phosphoglycerate mutase (F1NHM9); Phosphoserine aminotransferase (F1NTK1); Alpha-enolase (P51913); Fructose-bisphosphate aldolase (R4GM10); ATP-dependent 6-phosphofructokinase (A0A1D5PG85).
ko03010	Ribosome	Ribosomal protein (RP) L8 (F1NIX0); RPL19 (Q5ZKK8); 40S ribosomal protein S6 (P47838); RPL32 (E1BVB1); RPL24 (E1C8F7); RPL12 (E1BTG1); RPL4 (Q5ZII1); RPL35 (Q98TF7); RPL23A (E1BS06); Ubiquitin-60S ribosomal protein L40 (O42388); 40S ribosomal protein SA (P50890); RPL36a like (F1NIK4); 40S ribosomal protein S27 (A0A1D5NT52); RPL27A (A0A1D5PSU0); Mitochondrial ribosomal protein L10 (H9L0I5); RPS2 (E1C4M0); 40S ribosomal protein S8 (A0A1D5PT58); RPL6 (Q8UWG7); RPL13 (P41125); 40S ribosomal protein S27 (A0A1D5NZ06); RPL14 (A0A1D5PSZ9); RPS4 (P47836); Ubiquitin-40S ribosomal protein S27a (P79781); RCJMB04_19g1 (Q5ZJC1); RPS10 (E1C4N0); RPL15 (F1NQG5); RPL7 (Q5ZJ56).
ko00010	Glycolysis	Triosephosphate isomerase (P00940); Fructose-bisphosphate aldolase B (P07341); Fructose-bisphosphate aldolase (R4GM10); Phosphoglycerate kinase (P51903); Glyceraldehyde-3-phosphate dehydrogenase (F1NH87); ADH6 (A0A1D5P3A4); ATP-dependent 6-phosphofructokinase (Q90YA3); Alpha-enolase (P51913); Phosphoglycerate mutase (F1NHM9); Glucose-6-phosphate isomerase (Q5ZMU3); Phosphoglycerate mutase 1 (Q5ZLN1); Pyruvate kinase PKM (P00548); ATP-dependent 6-phosphofructokinase (A0A1D5PG85); Phosphoenolpyruvate carboxykinase [GTP] (P21642); Acetyltransferase component of pyruvate dehydrogenase complex (E1C6N5); Alcohol dehydrogenase (Q5ZK84).

Table S2. Statistics of KEGG enrichment in supplementary figure 2 (b)

Pathway ID	Pathway name	Protein name (Uniprot ID)
ko04141	Protein processing in endoplasmic reticulum	Endoplasmic reticulum chaperone BiP (Q90593); HSPA (Hsp70) binding protein 1 (A0A1D5P628); STIP1 homology and U box-containing protein 1 (Q5ZHY5); Eukaryotic translation initiation factor 2 subunit 1 (Q5ZLX2); Heat shock protein HSP 90-alpha (P11501); Cullin 1 (A0A1D5P2L3); DnaJ heat shock protein family (Hsp40) member C10 (E1BRA6); RCJMB04_2d15 (Q5ZMF5); Ubiquitin fusion-degradation 1-like protein (Q98UC3); DnaJ heat shock protein family (Hsp40) member A2 (Q5ZIZ7); SEL1L, ERAD E3 ligase adaptor subunit (E1C6X2); Prolactin regulatory element-binding protein (A0A077KY10); Valosin containing protein (Q5ZMU9); CANX (A0A1D5PF08); NSFL1 cofactor p47 (Q5ZK10); Dolichyl-diphosphooligosaccharide-protein glycosyltransferase subunit 1 (A0A1L1RTY4); Protein disulfide-isomerase A4 (Q5ZK20); Protein transport protein Sec23A (Q5ZK03); RCJMB04_2p21 (Q5ZM63); DnaJ heat shock protein family (Hsp40) member A1 (Q5ZJV3); RCJMB04_15d24 (Q5F3J8); SEC61A2 (A0A1D5NXC6); Sec61 translocon alpha 1 subunit (F1NBN1); BAG2 (R4GL30); Dolichyl-diphosphooligosaccharide-protein glycosyltransferase 48 kDa subunit (A0A1L1RYD3); Protein transport protein SEC23 (A0A1D5P0E2); Phospholipase A2 activating protein (A0A1D5NTN7).
ko00710	Carbon fixation in photosynthetic organisms	Fructose-bisphosphate aldolase (R4GM10); Triosephosphate isomerase (P00940); Phosphoglycerate kinase (P51903); Fructose-bisphosphate aldolase B (P07341); Glyceraldehyde-3-phosphate dehydrogenase (P00356); Malate dehydrogenase (E1BVT3); Transketolase (F1P1A5); Aspartate aminotransferase (P00508).
ko00071	Fatty acid	ACAT2 (F1NT20); Enoyl-CoA hydratase and 3-hydroxyacyl CoA dehydrogenase (E1C1T9); RCJMB04_26g6 (Q5ZIG7); RCJMB04_4j7 (Q5ZLW8); ADH6 (A0A1D5P3A4); HADHA (F1NI29); ACAA1 (F1NB64); ACAT1 (E1C0Q5); Acyl-CoA synthetase long chain family member 1 (Q5F420); Carnitine palmitoyltransferase 1A (Q6B842); RCJMB04_12m (17Q5ZK70).
ko00020	Citrate cycle	Isocitrate dehydrogenase (F1NPG2); Pyruvate carboxylase (Q8JHF6); Isocitrate dehydrogenase (Q5ZL82); Succinate dehydrogenase flavoprotein subunit (Q9YHT1); Aconitate hydratase (Q8AYI3); Succinate dehydrogenase flavoprotein subunit (F1NPJ4); Acetyltransferase component of pyruvate dehydrogenase complex (E1C6N5); Malate dehydrogenase (E1BVT3); Succinate dehydrogenase iron-sulfur subunit (A0A1D5PIF0); Phosphoenolpyruvate carboxykinase (P21642).
ko00720	Carbon fixation pathways in prokaryotes	Pyruvate carboxylase (Q8JHF6); ACAT1 (E1C0Q5); Isocitrate dehydrogenase (Q5ZL82); Isocitrate dehydrogenase (F1NPG2); Aconitate hydratase (Q8AYI3); ACAT2 (F1NT20).
ko01212	Fatty acid metabolism	Hydroxysteroid 17-beta dehydrogenase 12 (A0A1D5PPX3); Acyl-CoA synthetase long chain family member 1 (Q5F420); RCJMB04_26g6 (Q5ZIG7); RCJMB04_4j7 (Q5ZLW8); ACAA1 (F1NB64); ACAT2 (F1NT20); Enoyl-CoA hydratase and 3-hydroxyacyl CoA dehydrogenase (E1C1T9); HADHA (F1NI29); RCJMB04_12m17 (Q5ZK70); ACAT1 (E1C0Q5); Carnitine palmitoyltransferase 1A (Q6B842); Delta-6 fatty acid desaturase (A6NAB8)
ko00680	Methane metabolism	Fructose-bisphosphate aldolase (R4GM10); Phosphoserine aminotransferase (F1NTK1); Alpha-enolase (P51913); Fructose-bisphosphate aldolase B (P07341); Phosphoglycerate mutase (F1NHM9); ATP-dependent 6-phosphofructokinase (Q90YA3) ; ATP-dependent 6-phosphofructokinase (A0A1D5PG85); Phosphoglycerate mutase 1 (Q5ZLN1).
ko00970	Aminoacyl-tRNA biosynthesis	RCJMB04_13p14 (Q5ZK08); Tyrosine-tRNA ligase (Q5ZJ08); Lysine-tRNA ligase (Q5ZKP8); Glycyl-tRNA synthetase (Q5ZHR4); RCJMB04_4k14 (Q5ZLW1); Arginine-tRNA ligase (Q5ZM11); RARS2 (E1C229); RCJMB04_22h1 (Q5F3C0)

Table S3. Statistics of KEGG enrichment in supplementary figure 2 (c)

Pathway ID	Pathway name	Protein name (Uniprot ID)
ko04210	Apoptosis	Eukaryotic translation initiation factor 2 subunit 1 (Q5ZLX2); Inhibitor of apoptosis protein 3 (Q8UVF8); Inhibitor of nuclear factor kappa-B kinase subunit alpha (Q5ZJB4); Cathepsin D (Q05744); Tubulin alpha chain (A0A1D5NXV1); RAF proto-oncogene serine/threonine-protein kinase (P05625); Lamin-B2 (P14732); Lamin-A (P13648); Actin, cytoplasmic 2 (Q5ZMQ2); Cathepsin Z (E1C4M3); Tubulin alpha chain (A0A1D5P198); Cathepsin B (A0A1D5PUR5); Actin, cytoplasmic type 5 (P53478); Lamin-B1 (P14731); Tubulin alpha chain (A0A1D5PAR5); RCJMB04_35l12 (Q5ZHL7); BIRC2 (A0A140T8H6); Actin, cytoplasmic 1 (P60706).
ko00620	Pyruvate metabolism	Malate dehydrogenase (E1BVT3); Phosphoenolpyruvate carboxykinase (P21642); ACAT1 (E1C0Q5); Acetyltransferase component of pyruvate dehydrogenase complex (E1C6N5); ACAT2 (F1NT20); Pyruvate carboxylase (Q8JHF6); Pyruvate kinase PKM (P00548).
ko03018	RNA degradation	CCR4-NOT transcription complex subunit 7 (Q5ZJV9); ATP-dependent 6-phosphofructokinase (A0A1D5PG85); 60 kDa heat shock protein (Q5ZL72); RCJMB04_20e14 (Q5ZJ72); Polyribonucleotide nucleotidyltransferase 1 (A0A1D5NZ09); Stress-70 protein (Q5ZM98); CCR4-NOT transcription complex subunit 8 (Q5ZKA9); CNOT9 (A0A1D5PVC4); ATP-dependent 6-phosphofructokinase (Q90YA3); Alpha-enolase (P51913); Polyadenylate-binding protein (Q5ZL53).
ko04612	Antigen processing and presentation	MHC class II antigen (Q3HM56); CANX (A0A1D5PF08); Heat shock protein family A (Hsp70) member 4 (A0A1D5PPF8); Cathepsin B (A0A1D5PUR5); RCJMB04_2d15 (Q5ZMF5); TAP binding protein (A4F5B6); Heat shock protein HSP 90-alpha (P11501); Legumain (E1C958); Endoplasmic reticulum chaperone BiP (Q90593)
ko03013	RNA transport	Eukaryotic translation initiation factor 3 subunit D (F1NCE1); Eukaryotic translation initiation factor 2 subunit 1 (Q5ZLX2); Eukaryotic translation initiation factor 3 subunit H (Q5ZLE6); eIF4G-related protein NAT1 (Q5KTT9); XPOT (E1C593); Serine-threonine kinase receptor-associated protein (Q5ZL33); Polyadenylate-binding protein (Q5ZL53); Nuclear pore complex protein Nup93 (A0A1D5PFL9); Elongation factor 1-alpha 1 (Q90835); Nuclear cap-binding protein subunit 1 (Q5ZJZ6); Eukaryotic translation initiation factor 3 subunit F (E1C050); Nucleoporin 98 (A0A1D5PBD8); Eukaryotic initiation factor 4A-II (Q8JFP1); Nuclear pore complex protein Nup85 (Q5ZIU6); Ribonucleic acid export 1 (E1C7F8); RCJMB04_19c24 (Q5ZJD2).
ko04146	Peroxisome	Acyl-CoA synthetase long chain family member 1 (Q5F420); Peroxisomal targeting signal 1 receptor (Q5ZMQ9); RCJMB04_26g6 (Q5ZIG7); Enoyl-CoA hydratase 1 (A0A1D5PDA1); Isocitrate dehydrogenase (Q5ZL82); ABCD2 (A0A1D5PHR1); Peroxiredoxin-1 (P0CB50); ATP binding cassette subfamily D member 3 (Q5F4B9); ACAA1 (F1NB64); Isocitrate dehydrogenase (F1NPG2); Enoyl-CoA hydratase and 3-hydroxyacyl CoA dehydrogenase (E1C1T9).
ko00051	Fructose and mannose metabolism	Triosephosphate isomerase (P00940); Fructose-bisphosphate aldolase B (P07341); Fructose-bisphosphate aldolase (R4GM10); ATP-dependent 6-phosphofructokinase (Q90YA3); ATP-dependent 6-phosphofructokinase (A0A1D5PG85); Phosphoglycerate kinase (P51903).
